# Effect of sarcopenia on refractures of adjacent vertebra after percutaneous kyphoplasty

**DOI:** 10.1186/s12891-024-07295-3

**Published:** 2024-03-12

**Authors:** Chengnan Jing, Huazheng Wang, Peng Liu, Shaofeng Yang, Linlin Zhang, Peng Yang, Minfeng Gan

**Affiliations:** The Department of Orthopaedics, the First Affiliated Hospital of Soochow University, 899 Pinghai Road, Suzhou, 215006 Jiangsu China

**Keywords:** Sarcopenia, Spinal fracture, Percutaneous kyphoplasty, Refracture

## Abstract

**Purpose:**

To explore the effect of sarcopenia on recurrent fractures of adjacent vertebra after percutaneous kyphoplasty (PKP).

**Methods:**

A total of 376 osteoporotic vertebral compression fractures (OVCFs) patients over 55 years old who were admitted to the Hospital from August 2020 to January 2021 were selected. Among them, 38 patients with recurrent fractures in adjacent vertebra after PKP were selected as the refracture group (RG), and the remaining 338 patients were selected as the non-refracture group (NRG). The age, gender, grip strength, body mass index (BMI), bone mineral density (BMD), visual analogue scale (VAS) of pain before and one month after surgery, Oswestry disability index (ODI) before and one month after surgery and the occurrence of sarcopenia were compared between the two groups. Logistic regression analysis was used to evaluate the effect of related risk factors on refracture after vertebral PKP.

**Results:**

The results of t-test and Chi-square test showed that there were no obvious differences in gender, BMI, preoperative VAS score (t=-0.996, *P* = 0.320) and ODI (t=-0.424, *P* = 0.671), one month postoperative VAS score (t=-0.934, *P* = 0.355) and ODI score (t=-0.461, *P* = 0.645). while the age and grip strength showed significant differences between the two groups. Logistic regression analysis showed that BMI and gender had no significant effect on refracture after PKP, while sarcopenia and advanced age were independent risk factors for refracture after PKP. Also, increased BMD was a protective factor for refracture after PKP.

**Conclusion:**

Sarcopenia is an independent risk factor for recurrent fractures after PKP in OVCF patients. The screening and diagnosis of sarcopenia should be strengthened. At the same time, anti-sarcopenia treatment should be actively performed after surgery.

## Background

With the development of the social economy and medical security system, the degree of aging is constantly developing, and the incidence of osteoporotic vertebral compression fractures also shows a trend of increasing [1]. At present, percutaneous kyphoplasty is usually the main method of treatment for osteoporotic vertebral compression fractures(OVCFs) patients. percutaneous kyphoplasty(PKP) can relieve symptoms, restore vertebral height and improve the prognosis. However, for patients with OVCF, PKP can only improve the diseased vertebrae, and restore the height of the vertebral body [2–4]; due to the loss of bone mass in patients, PKP is difficult to effectively prevent vertebra fractures adjacent to the diseased vertebrae. Therefore, refracture of adjacent vertebra after PKP is still an urgent problem to be solved [5]. Sarcopenia is a progressive and systemic disorder that primarily refers to the destruction of skeletal muscle shape and function, it is characterized by the loss of muscle mass and quantity, and leads to a number of adverse outcomes, including increased risk of falls, decreased limb function, physical weakness or even death [6]. Some researchers have pointed out that sarcopenia is closely related to the occurrence of osteoporosis [7, 8], so the effect of sarcopenia on refractures of adjacent vertebra in patients with OVCF deserves further investigation. This study is based on the study of patients with refractures of adjacent vertebra after PKP, and further explores the effect of sarcopenia on refractures of adjacent vertebra, in order to improve the prevention strategy of OVCF high-risk groups and prevent the occurrence of refractures.

## Methods

1. This study retrospectively analyzed OVCF patients over 55 years old who were admitted to the First Affiliated Hospital of Soochow University from August 2020 to January 2021. This study was approved by the Ethics Committee of the First Affiliated Hospital of Soochow University. Inclusion criteria: 1)age ≥ 55 years; 2)Vertebral compression fracture (including instability of vertebral body, fresh fracture, delayed union) ; 3)patients with PKP; 4)BMD T value <-2.5SD; 5)refusal to stay in bed for conservative treatment; 6)no congenital lower limb deformity or dysfunction; Exclusion criteria: 1)patients with spinal cord injury; 2)vertebral fractures caused by violent violence; 3)neurological diseases affecting limb sensation and function; 4)severe cardiovascular or cerebrovascular diseases, and poorly controlled malignant hypertension; 5)diabetes with complications; 6)refusal of surgery or intolerance Recipients; 7)primary or metastatic tumors of the spine. According to the inclusion and exclusion criteria, a total of 376 patients were included in this study, and they were divided into 2 groups according to whether they had recurrent fractures after PKP. There were 38 cases in the refracture group (RG) and 338 cases in the non-refracture group (NRG). There were 71 males and 305 females. Their mean age was 70.37. Among them, 32 patients had confirmed diagnoses of sarcopenia in RG, and 145 patients in NRG were diagnosed as sarcopenia. Besides, all the patients have received anti-osteoporotic therapy, including the use of Calcium, vitamin D, denosumab and bisphosphonate since their first fracture.

### Diagnostic criteria and surgery

#### Diagnosis

**Fig. 1:**
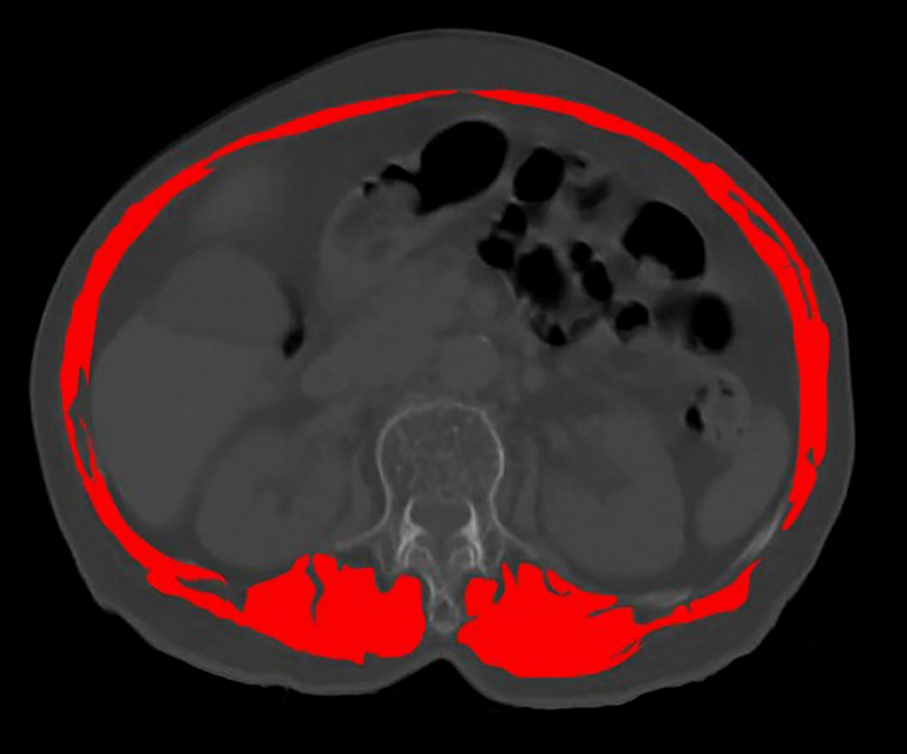
Calculate the muscle area On the CT image at the level of the pedicle of the thoracic 12 vertebrae

The diagnostic criteria for sarcopenia refer to the latest guidelines [6] recommended by the European Sarcopenia Working Group(EWGSOP) in 2019:grip strength :male<27 kg, female<16 kg; For the patients with reduced grip strength, we calculated the skeletal muscle indices (SMI), which sum measured by muscle area divided by the square of the patient’s height. Muscle area calculation method: On the CT image at the level of the pedicle of the thoracic 12 vertebrae, the measurement includes the erector spinae, latissimus dorsi, internal oblique, external oblique, rectus abdominis, external intercostal muscle and intercostal muscle (Fig. 1). The diagnostic value for SMI at the thoracic 12 level proposed by Nemec [9] et al.:<42.6cm^2^/m^2^(male) and<30.6cm^2^/m^2^(female). All data were independently measured by two physicians with more than 3 years of work experience. The patient’s grip strength and SMI value were both less than the diagnostic value to be diagnosed as sarcopenia, otherwise it was diagnosed as non-sarcopenia. The bone mineral density(BMD) was detected using dual-energy X-rays, we measured the bone density of L1-L4 and then averaged it.

### Percutaneous kyphoplasty

After successful anesthesia and sterilization, we positioned the puncture point, and then hammered into the vertebra with the puncture needle; after the working cannula was placed; the balloons were also placed through the working cannula, then we expanded the balloon. After the bone cement was prepared, the balloon was taken out and push into the bone cement through the working cannula. X-ray showed that the bone cement was well distributed in the vertebra and the operation was completed.

### Evaluation and follow-up

Basic information such as age, gender, height, weight, BMD and sarcopenia were recorded. The main indicators for evaluating the efficacy of surgery included: preoperative VAS [10, 11] score and ODI [12], postoperative VAS score and ODI one month after operation.

### Statistics analysis

The t-test and Chi-square test was used to analyze the general conditions and surgery-related indicators of the patients in the refracture group and the non-refracture group. At the same time, logistic regression analysis was used to clarify the influence of gender, age, BMI, BMD and sarcopenia on refractures of adjacent vertebra after PKP.SPSS21(IBM Corp., Armonk, NY, USA) was used for statistical processing. Continuous variables were expressed as “mean ± standard deviation”, *P* < 0.05(★) was considered statistically significant, and “ns” was used to indicate that there is no statistical difference.

## Results

**Fig. 2:**
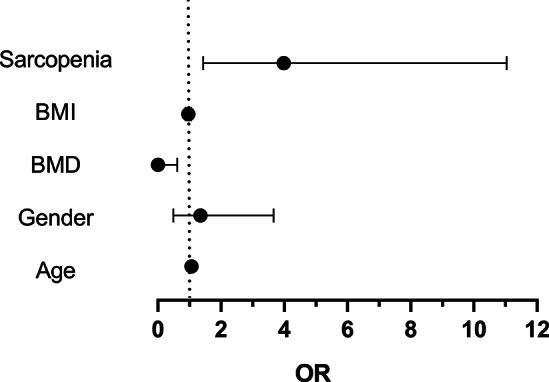
Results of the logistic regression analysis for sarcopenia, BMI, BMD, gender and age

1. The results of t-test and Chi-square test showed that there were no obvious differences in gender(X^2^ = 0.264, *P* = 0.607) and BMI(t=-1.726,*P* = 0.085), while the age(t = 4.560,*P* = 0.001) and grip strength(t=-10.247,*P* = 0.001) showed significant differences between the two groups (Table 1). Besides, preoperative VAS score (t=-0.996, *P* = 0.320) and ODI (t=-0.424, *P* = 0.671), one month postoperative VAS score(t=-0.934, *P* = 0.355) and ODI score(t=-0.461,*P* = 0.645) (Table 1).
2. Logistic regression analysis was performed on data such as gender, age, BMI, BMD, and sarcopenia (Table 2). The results showed that gender and BMI had no significant effect on refracture after PKP, while sarcopenia and advanced age were independent risk factors for refracture after PKP. Also, increased BMD was a protective factor for refracture after PKP (Fig. 2).

**Table 1:** The baseline characteristics of the study population and the evaluation of the efficacy of surgery

	RG	NRG	t	P
Age	76.21 ± 8.82	69.71 ± 8.28	4.560	0.001
BMI	23.54 ± 3.87	24.78 ± 4.25	-1.726	0.085
Grip strength	13.87 ± 1.77	17.37 ± 3.40	-10.247	0.001
preoperative VAS	4.47 ± 1.16	4.69 ± 1.26	-0.996	0.320
postoperative VAS	1.79 ± 1.09	1.96 ± 0.90	-0.934	0.355
Preoperative ODI	59.53 ± 9.02	60.18 ± 9.05	-0.356	0.722
Postoperative ODI	44.63 ± 8.47	45.22 ± 8.12	-0.461	0.645

**Table 2:** The exact value of the logistic regression analysis

Risk factors	Age	Gender	BMD	BMI	Sarcopenia
OR	1.063	1.344	0.003	0.960	3.981
95%CI	1.011–1.118	0.493–3.665	0.001–0.615	0.875–1.054	1.436–11.041

**Fig. 3:**
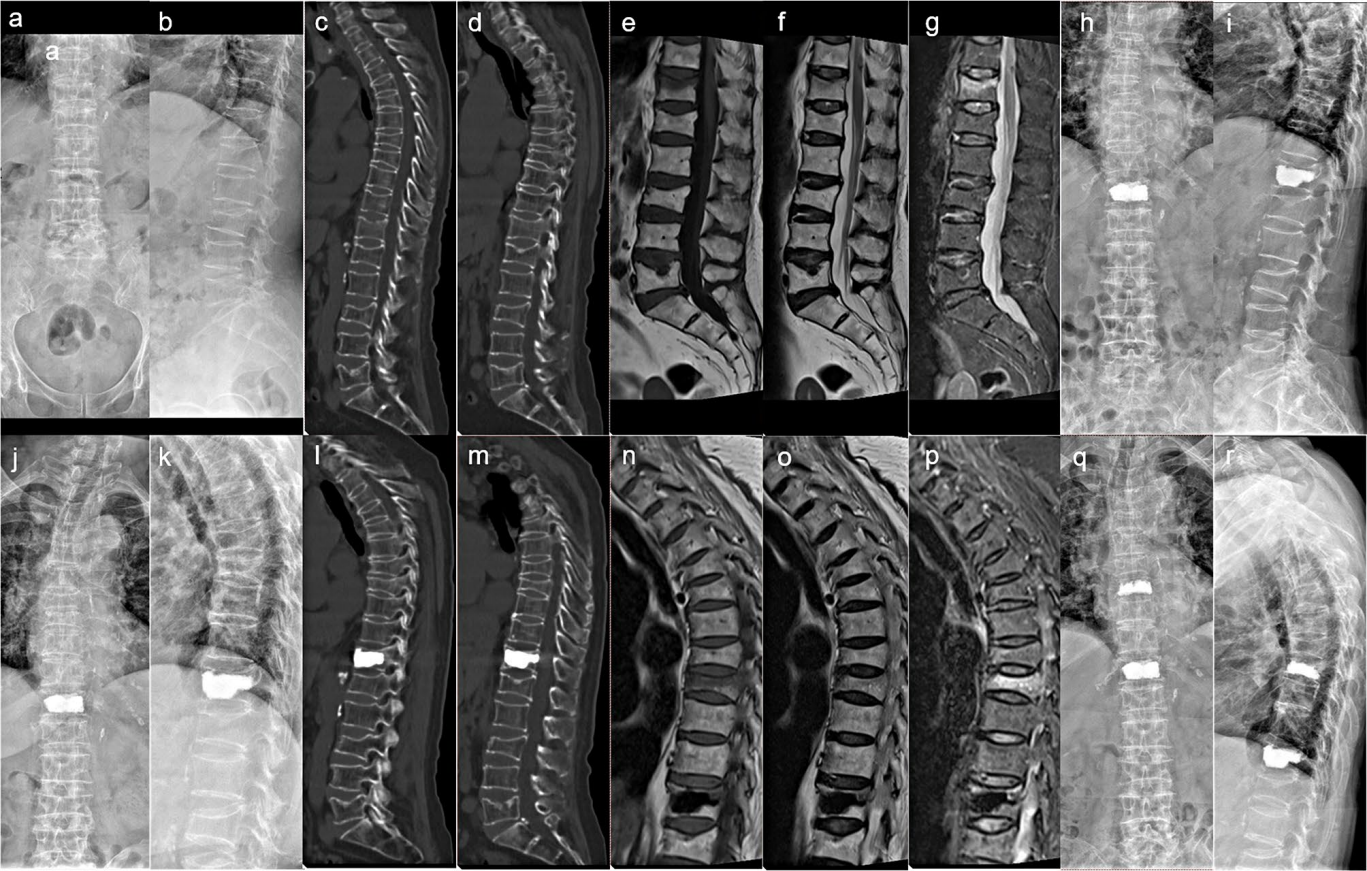
A typical case: a 76 years old women who was diagnosed as sarcopenia got OVCF twice within one year

3. Here is a typical case. It was a woman who is 76 years old. Figure 3a and g showed her first OVCF in T12 before KP, and during her hospitalization, she was diagnosed as sarcopenia; Fig. 3h and i showed the Post-operative review results. Then Fig. 3j and p showed her second OVCF in T9 before the surgery, and Fig. 3q and r showed the follow-up results 3 months after the surgery (Fig. 3).

## Discussion

Sarcopenia was first proposed by Rosenberg [13] in the 1980s, it is a degenerative disease characterized by a decrease in the relative mass of skeletal muscle and decreased skeletal muscle strength. Sarcopenia currently occurs in 4.4-23% of people aged 65 and older worldwide [14–16]. According to the 2019 report of the Asian Sarcopenia Working Group (AWGS), the prevalence of sarcopenia in the elderly population in Asia is 5.5-25.7%, of which the prevalence of males is 5.1-21.0%, while the prevalence of females is 4.1 -16.3% [17]. In this study, we found that the BMI of sarcopenic patients is usually lower than that of non-sarcopenic patients, but there is no clear correspondence between them, that means patients with the same BMI may have a huge difference in SMI, which suggests that the skeletal muscle level of patients cannot be screened only by BMI.

Osteoporosis (OP) is the most common bone disease, which is mainly characterized by decreased bone mass, damage to the microstructure of bone tissue, increased bone fragility, and prone to fractures [18]. Common sites of osteoporotic fractures include vertebra, hip, distal forearm, proximal humerus, and pelvis, among which vertebra fractures are the most common [19]. For patients with OVCFs, PKP has become the first choice for many patients due to its rapid symptom relief, good vertebral height recovery, short operation time and lower price. In this study, the postoperative VAS and scores of the patients were significantly improved compared with those before the operation, which also confirmed the good effect of PKP on pain relief and functional recovery. However, since the patient’s osteoporosis has not been fundamentally improved, refractures of adjacent vertebra after PKP occur frequently. Nolan et al. also proposed that the occurrence of osteoporosis significantly increases the risk of recurrent fractures of adjacent vertebral bodies after PKP [20].

Since the concept of sarcopenia was proposed in 1989, researchers have gradually discovered the influence of metabolic factors, genetic factors and environmental factors on sarcopenia. Hirschfeld [21] et al. proposed that, based on the infiltration of adipose tissue into bone and muscle, obese patients usually need to consider the possibility of both osteoporosis and sarcopenia. The basic research on sarcopenia also pointed out that the relationship between bone and muscle not only has mechanical interaction, but also affects each other’s physiological processes through endocrine and paracrine pathways [22, 23]. This allows us to further understand the relationship between osteoporosis and sarcopenia. In the present study, sarcopenia was closely associated with refractures after PKP in OVCF patients. Of the 376 patients, 38 had recurrent fractures in adjacent vertebra after PKP. We performed logistic regression analysis on these 376 cases and found that sarcopenia significantly increased the risk of refractures in adjacent vertebra after PKP. Therefore, clinicians should pay attention to formulating an individualized plan for patients after PKP, strengthen the screening of sarcopenia, and concentrate on the supplementation of limb function exercise and nutritional diet. In this study, although we have analyzed some potential risk factors, the amount is limited. Some risk factors, such as claudication and history of glucocorticoid use, were needed a further comparison between two groups and a longer period of follow-up.

## Conclusions

Sarcopenia is an independent risk factor for recurrent fractures after PKP in OVCF patients; it is necessary to strengthen the screening and diagnosis of sarcopenia. and perform anti-sarcopenia treatment after surgery, so as to reduce the risk of refractures.

## Data Availability

The datasets used and analyzed during the current study are available from the corresponding author on reasonable request.

## Abbreviations

PKP: percutaneous kyphoplasty
OVCF: osteoporotic vertebral compression fractures
RG: refracture group
NRG: non-refracture group
BMI: body mass index
BMD: bone mineral density
VAS: visual analogue scale
ODI: Oswestry disability index
SMI: skeletal muscle indices
